# Spontaneously Ruptured Pancreatic Mucinous Cystic Neoplasm: A Case Report

**DOI:** 10.70352/scrj.cr.24-0087

**Published:** 2025-02-01

**Authors:** Masataka Hirano, Masanori Tsujie, Takayoshi Goto, Chikato Koga, Soichiro Mori, Daisuke Takiuchi, Kentaro Nishida, Masatoshi Nomura, Yukihiro Yoshikawa, Koki Tamai, Takuya Hamakawa, Mitsuyoshi Tei, Yusuke Akamaru

**Affiliations:** 1 Department of Gastroenterological Surgery, Osaka Rosai Hospital, Sakai, Osaka, Japan; 2 Department of Diagnostic Pathology, Osaka Rosai Hospital, Sakai, Osaka, Japan

**Keywords:** pancreas, mucinous cystic neoplasm, MCN, rupture, distal pancreatectomy

## Abstract

**INTRODUCTION:**

Pancreatic mucinous cystic neoplasm (MCN) is a cystic tumor of the pancreas typically located in the pancreatic body or tail in middle-aged women. However, MCN rupture is rare. This report describes a case of MCN with spontaneous rupture during follow-up.

**CASE PRESENTATION:**

The patient was a 34-year-old woman. Contrast-enhanced computed tomography (CECT) and magnetic resonance imaging (MRI) revealed a 130 mm multifocal cyst in the pancreatic tail. The cyst, characterized by multiple septa and cyst-in-cyst structures, was diagnosed as an MCN. Initially, the patient opted for periodic follow-ups instead of surgical resection. After a gradual increase in cyst size, surgery was scheduled approximately 1 year later. Two days before the scheduled surgery, the patient experienced unexplained lower abdominal pain. Moreover, CECT revealed a shrinking cystic mass in the pancreatic tail along with the presence of ascites, leading to a diagnosis of spontaneous rupture of the pancreatic cyst. No peritonitis was detected, and a distal pancreatectomy was performed 2 days after admission. Pathological examination confirmed that the pancreatic cyst was a noninvasive mucinous cystadenocarcinoma. The abdominal cavity contained large amounts of turbid ascites with neutrophils but no bacterial growth. Strong inflammatory changes were noted at the cyst wall disruption site. Despite the development of a pancreatic fistula (ISGPF Grade BL, Clavien–Dindo Grade II), the patient was discharged from the hospital on postoperative day 16 and remained alive and recurrence-free for 18 months after surgery.

**CONCLUSION:**

Spontaneous rupture of an MCN is rare. In this study, we report our case and review previously published cases of MCN rupture. We also discuss the potential causes of the spontaneous rupture in our case.

## Abbreviations

CECT
: contrast-enhanced computed tomography
DP
: distal pancreatectomy
ISGPF
: International Study Group on Pancreatic Fistula Definition
MCA
: mucinous cystadenoma
MCC
: mucinous cystadenocarcinoma
MCN
: mucinous cystic neoplasm
MRI
: magnetic resonance imaging
OTS
: ovarian-type stroma
PD
: pancreaticoduodenectomy
PgR
: progesterone receptor
SPDP
: spleen-preserving distal pancreatectomy

## INTRODUCTION

Mucinous cystic neoplasms (MCNs) are polycystic pancreatic tumors lined with mucin-secreting epithelial cells and characterized by ovarian-type stroma (OTS). MCNs are almost exclusively located in the body or tail of the pancreas in middle-aged women.^1)^ MCN rupture is rare due to the presence of a thick fibrous capsule.^2)^ In this report, we present a case of spontaneous rupture of the MCN without any specific trigger and describe our approach, including distal pancreatectomy (DP).

## CASE PRESENTATION

**Fig. 1:**
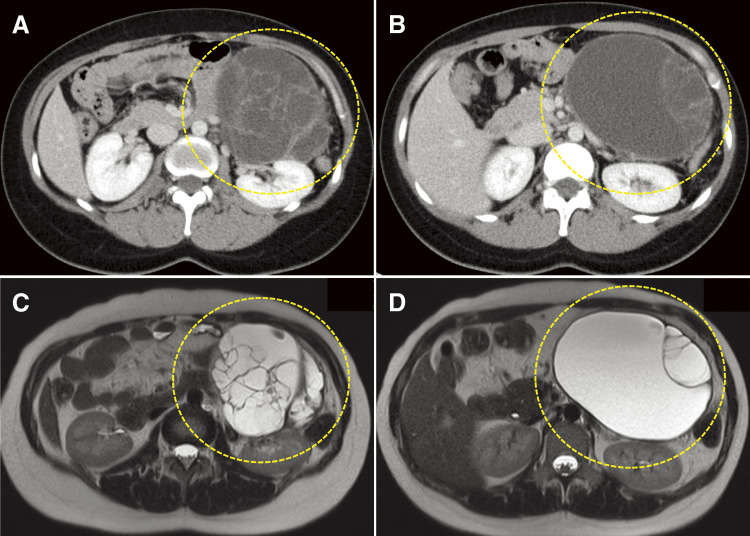
Imaging findings at the time of initial examination: (**A, B**) CECT. A multifocal cystic mass with a length of 130 mm in the pancreatic tail with multiple septa and a cyst appearance (dotted line) consistent with a pancreatic MCN. (**C, D**) T2-weighted magnetic resonance images. The cyst content had signal intensities akin to those of water. Although some of the septal walls appear thick, no mural nodules are observed. CECT, contrast-enhanced computed tomography; MCN, mucinous cystic neoplasm

A previously healthy 34-year-old woman with no significant medical history presented to her physician with the chief complaint of epigastric discomfort. Although the patient’s symptoms improved with medical treatment, abdominal ultrasonography revealed a 130 mm mass in the pancreatic tail. She was referred to the Department of Gastroenterology of our hospital for further evaluation. Contrast-enhanced computed tomography (CECT) showed a multifocal cystic mass with a length of 130 mm in the pancreatic tail, with multiple septa and a cyst appearance consistent with a pancreatic MCN. The mass compressed the stomach and colon but showed no significant hemorrhage, calcification, or main pancreatic duct dilatation (**Fig. 1**).

Magnetic resonance imaging (MRI) showed a multifocal cyst with a diameter of 130 mm bulging from the pancreatic tail to the left abdomen. T2-weighted images indicated that the cyst contents had signal intensities similar to water, with some septal walls appearing thick but no mural nodules. As with CECT findings, there was no significant dilatation of the main pancreatic duct or lymph node enlargement (**Fig. 1**).

**Fig. 2:**
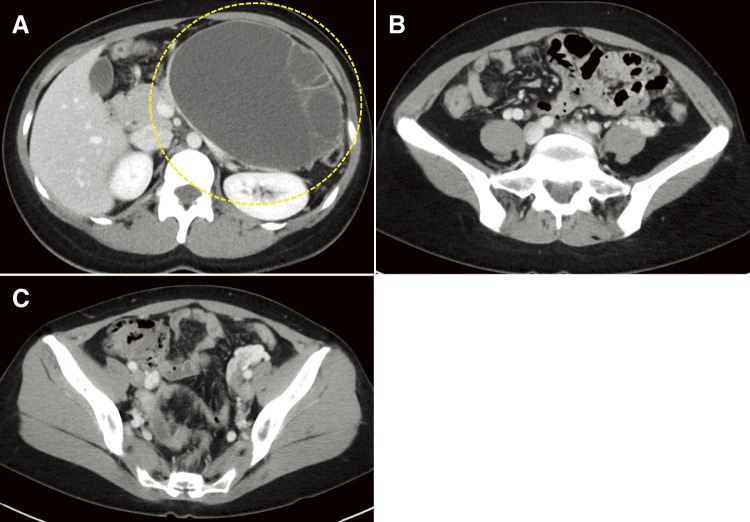
The CECT findings 10 months after the first examination: (**A**) A multifocal cystic mass measuring 150 mm in length was observed in the pancreatic tail (dotted line), demonstrating a tendency to enlarge mildly. (**B, C**) No ascites were observed. CECT, contrast-enhanced computed tomography

Based on these imaging test results, we suspected that the cyst was a pancreatic MCN. The patient was referred to our department for further surgical treatment. Despite our recommendation for surgery, she preferred close monitoring. Approximately 1 year after the initial diagnosis, she decided to undergo surgery owing to an increase in the cyst size (**Fig. 2**).

**Fig. 3:**
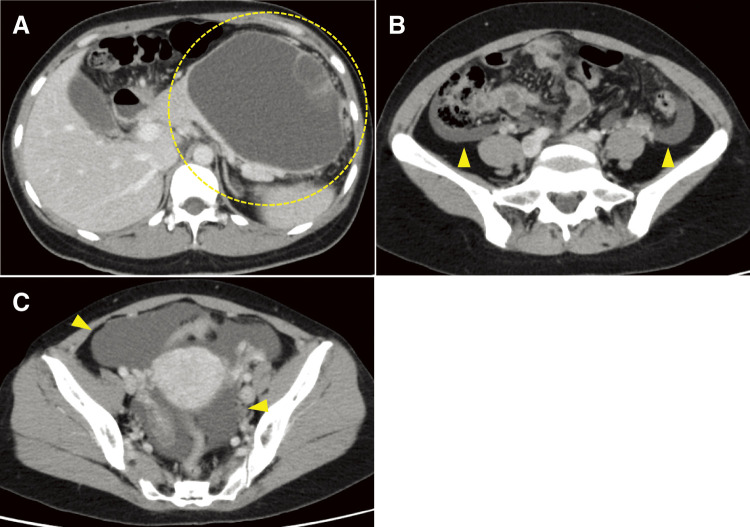
The CECT revealed findings upon the patient’s visit to our emergency department: (**A**) A shrinking cystic mass in the pancreatic tail (dotted line). (**B, C**) Ascites from the paracolonic groove to the pelvic region (arrowheads). CECT, contrast-enhanced computed tomography

Two days before the scheduled surgery, the patient presented to the emergency department with abdominal pain. She presented with spontaneous pain and tenderness in the upper abdomen but no peritoneal irritation symptoms. Blood tests showed mildly elevated inflammatory and pancreatic enzyme levels. CECT indicated a shrinking cystic mass in the pancreatic tail and ascites, suggesting a ruptured pancreatic cyst (**Fig. 3**). No trauma or other triggers were observed before the onset of abdominal pain, leading to the diagnosis of spontaneous rupture of the MCN. Considering her stable general condition without peritonitis or hemorrhage, a DP was performed on the initially planned day, 2 days after admission.

**Fig. 4:**
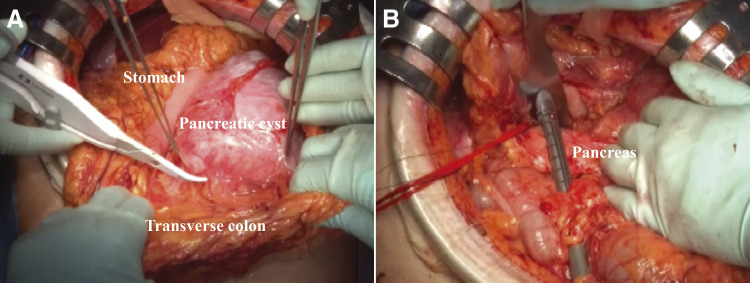
The surgical findings: (**A**) A huge mass is observed in the left upper abdomen, covered by greater omentum, with adhesions to the posterior wall of the stomach and transverse colon mesentery. (**B**) The pancreas was resected along the portal vein path using an endoscopic linear stapler.

**Fig. 5:**
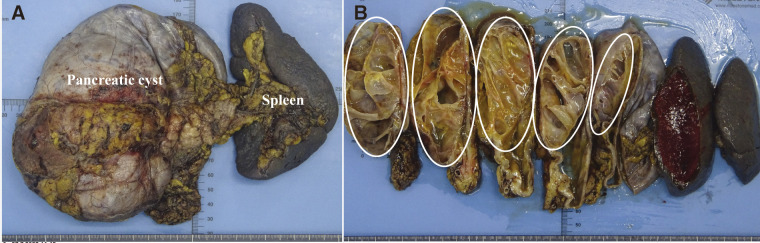
The gross findings of the excised specimen: (**A**) Pancreas and spleen. (**B**) The specimens included the distal pancreas and spleen, with a 150 mm-sized cystic tumor with multifocal interiors (dotted line).

Surgical findings included the operative time of 190 min and blood loss of 50 mL, with no requirement for transfusion. The abdominal cavity contained a large amount of turbid fluid. Rapid intraoperative cytological examination of the ascites was negative (CY0), and cultures showed no bacteria growth. A huge mass was observed in the left upper abdomen, covered by the greater omentum, with adhesions to the posterior wall of the stomach and transverse colon mesentery. The dorsal pancreas was tunneled via blunt dissection of the anterior surface of the portal vein from the inferior border of the pancreas. The splenic arteries and veins were ligated and dissected. The pancreas was resected along the portal vein path using an endoscopic linear stapler. The dorsal pancreas was dissected, the adhesions of the greater omentum and the colonic mesentery to the tumor were detached without damaging the mesenteric vessels, and the excised specimen revealed a 150 mm-sized cystic tumor with multifocal interiors (**Figs. 4** and **5**). A rupture hole, approximately 3 mm in diameter, was detected on the ventral side of the largest cyst. The fluid from the ruptured cyst was cloudy yellow, while the non-ruptured cyst fluid was clear with slightly yellowish mucus. Cytology of both fluids showed no malignant cells (class II), and cultures were negative.

**Fig. 6:**
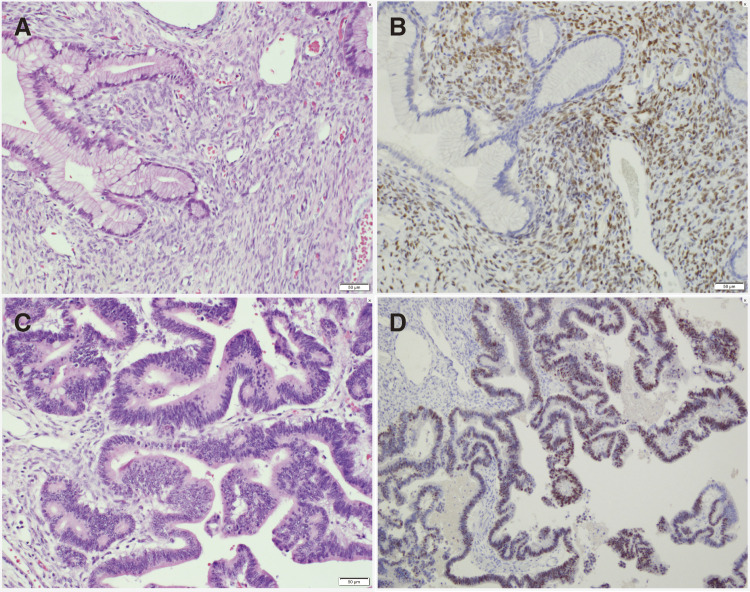
The macroscopic findings of the pancreatic cyst showed a multifocal tumor, exhibiting continuity to the pancreatic parenchyma. The tumor also demonstrated mucous epithelium and PgR-positive ovarian-type stroma. Papillary growth of p53-positive atypical columnar epithelium on some cyst walls was noted, but no evidence of stromal invasion or malignant findings in dissected lymph nodes was observed. (**A:** hematoxylin and eosin staining, 200×, **B:** progesterone receptor staining, hematoxylin and eosin stain, 200×, **C:** Hematoxylin and eosin staining, cancer department, 200×, **D:** p53 staining, cancer department, 200×). PgR, progesterone receptor

**Fig. 7:**
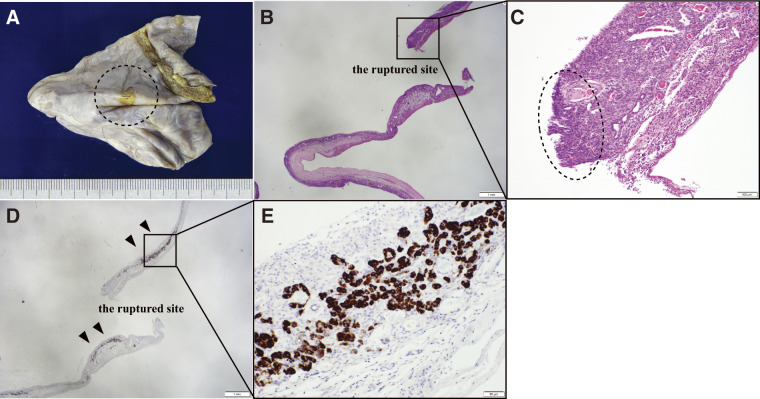
The findings at the site of the failed pancreatic cyst included a localized yellowish color change on the serosal side of the ruptured area (**A**, dotted line). Surrounding the ruptured site, the fibrous connective tissue layer was absent, and the cyst wall predominantly contained ovarian-type stroma components (**B**). The serosal side of the rupture exhibited strong inflammatory changes with desquamated epithelium and no malignant cells (**C**). BCL10 staining revealed many positive pancreatic acinar cells near the ruptured site (**D, E**). (**A**: gross findings, **B**: hematoxylin and eosin staining, 12.5×, **C**: hematoxylin and eosin staining, 100×, **D**: BCL10 staining, 12.5×, **E**: BCL10 staining, 200×).

Pathological examination revealed a multifocal tumor continuous with the pancreatic parenchyma, showing mucous epithelium and progesterone receptor (PgR)-positive OTS. Papillary growth of p53-positive atypical columnar epithelium was noted on some cyst walls, but no evidence of stromal invasion or malignancy in the dissected lymph nodes was observed. Based on these findings, the tumor was diagnosed as a noninvasive mucinous cystadenocarcinoma (MCC) (**Fig. 6**). Surrounding the rupture site, the fibrous connective tissue layer was absent, and the cyst wall predominantly contained OTS components. The serosal side of the rupture exhibited strong inflammatory changes with desquamated epithelium and no malignant cells. Furthermore, BCL10 staining revealed many positive pancreatic acinar cells near the rupture site (**Fig. 7**).

In the postoperative course, the patient experienced a pancreatic fistula (ISGPF Grade BL, Clavien–Dindo Grade II) and was discharged on postoperative day 16. She is currently undergoing outpatient follow-up and remains alive and recurrence-free 1 year and 6 months after surgery.

## DISCUSSION

MCNs are characterized by the presence of mucin-producing columnar epithelium and OTS.^1)^ Moreover, MCN accounts for 1% of pancreatic tumors and predominantly affects women aged 40–60 years. Typically, MCN does not affect the pancreatic duct and is localized in the pancreatic body and tail.^1)^ MCNs are classified according to the degree of dysplasia into mucinous cystadenoma, mucinous cystic tumors with moderate dysplasia, and noninvasive and invasive MCC.^3)^ This progression underscores the importance of surgical resection to prevent carcinomatous transformation.^4)^ Notably, MCC has been reported to have a better prognosis than does typical pancreatic ductal adenocarcinoma^5)^; however, invasive MCC has a poor prognosis and may cause peritoneal dissemination if cystic fluid leaks during manipulation.^1)^

The rarity of MCN rupture is attributed to its thick, fibrous capsules.^2)^ Our literature review, involving cases reported on PubMed between 1986 and 2023 using three keywords, “mucinous cystic neoplasm,” “pancreas,” and “rupture,” identified 11 cases excluding our case^6–16)^ (**Table 1**). All patients, including our case, were women with a median age of 33.5 (28–69) years. One case involved a pancreatic head treated with pancreaticoduodenectomy (PD), whereas others involving the pancreatic tail or body required DP. Seven patients were diagnosed with MCC, including ours. One of them was invasive, while the remaining six were noninvasive. The median follow-up duration was 20 (2–102) months. Two patients, including one with invasive MCC, experienced recurrence.^9,16)^ The patient with invasive MCC received four courses of gemcitabine therapy after surgery because of the ruptured case of malignant tumor. However, re-operation was performed because of mechanical ileus by the local recurrence of the tumor 7 months after initial surgery; then she received additional gemcitabine therapy (a total of 16 courses).^9)^ The other patient with noninvasive MCC was diagnosed with peritoneal dissemination 9 months after surgery. Gemcitabine and nab-paclitaxel therapy were administered. The lesion had almost disappeared 12 months after surgery, then chemotherapy continued.^16)^

**Table 1:** Reported cases of ruptured MCNs

No.	Author	Year	Age	Sex	Location	Tumor size (mm)	Surgical procedure	Diagnosis	Pregnant	Follow-up (months)	Recurrence	Outcome
1	Smithers^6)^	1986	33	F	Body/Tail	100	DP	MCC (noninvasive)	Yes	Unknown	Unknown	Unknown
2	Ozden^7)^	2007	32	F	Tail	150	SPDP	MCC (noninvasive)	Yes	12	No	Alive
3	Bergenfeldt^8)^	2007	42	F	Body	200	DP	MCA	No	19	No	Alive
4	Naganuma^9)^	2011	32	F	Head	110	PD	MCC (invasive)	Yes	36	Local	Alive
5	Imoto^10)^	2013	69	F	Body/Tail	60	DP	MCC (noninvasive)	No	2	No	Alive
6	Haddad^11)^	2019	30	F	Tail	150	DP	MCA	No	36	No	Alive
7	Fujinaga^12)^	2020	28	F	Tail	80	SPDP	MCA	No	96	No	Alive
8	Revoredo^13)^	2020	38	F	Body/Tail	200	DP	MCA	Yes	96	No	Alive
9	Krishnamurthy^14)^	2021	39	F	Body/Tail	92	DP	MCA	No	6	No	Alive
10	Tezuka^15)^	2022	28	F	Tail	90	DP	MCC (noninvasive)	No	102	No	Alive
11	Oyama^16)^	2023	53	F	Tail	75	DP	MCC (noninvasive)	No	20	Peritoneal dissemination	Alive
12	Our case	2023	34	F	Tail	150	DP	MCC (noninvasive)	No	18	No	Alive

DP, distal pancreatectomy; MCA, mucinous cystadenoma; MCC, mucinous cystadenocarcinoma; MCN, mucinous cystic neoplasm; PD, pancreaticoduodenectomy; SPDP, spleen-preserving distal pancreatectomy

Four cases involved pregnant women.^6,7,9,13)^ Regardless of the origin, this stroma is hormone-sensitive and regularly expresses PgRs.^17)^ In other words, the development of MCN in women and the rapid growth of MCN during pregnancy indicate a significant role for sex hormones in MCN tumorigenesis.^18)^

No study has elucidated the cause of MCN rupture other than the involvement of sex hormones during pregnancy. We discuss the cause of MCN rupture in our case. Our case, as well as generally observed MCNs, had a robust fibrous capsule. Although the MCN was found to increase with time before the rupture, and the traction of the cyst wall is attributed to cyst growth, the robust fibrous capsule of the MCN makes it unlikely that this factor alone causes perforation. Furthermore, no history of trauma preceded the presentation of abdominal pain, ruling out external trauma as the cause. Additionally, cancer invasion was also excluded as no evidence of malignancy near the site of cyst rupture was observed. Under these circumstances, pathological examination revealed the disappearance of the fibrous connective tissue layer around the rupture site, accompanied by significant inflammatory changes on the serosal side. Therefore, we speculated that the cyst rupture in our case was caused by inflammatory changes in the cyst wall, leading to its weakening.

To further investigate the cause of these inflammatory changes, we performed a culture examination of the ascitic fluid and cyst contents, which ruled out bacterial or fungal infections as direct causes of inflammation. Additionally, BCL10 staining, specific to pancreatic acinar cells, was conducted,^19)^ revealing a notable presence of positioned cells and conduits formed by these cells near the rupture site. This indicates that exocrine secretion, specifically pancreatic juice, was present.

Based on these findings, it might be possible that the rupture in our case was caused by a combination of cyst wall traction due to cyst growth and localized inflammatory changes. The inflammation might have resulted from the stasis of pancreatic fluid, leading to tissue fragility due to circulatory failure and localized pancreatitis. These findings contribute to our understanding of the mechanisms underlying ruptured MCN.

## CONCLUSION

Ruptured MCNs are rare. In this study, we demonstrated for the first time that inflammation associated with pancreatic fluid stasis may be one of the causes of MCN rupture.

## ACKNOWLEDGMENTS

We would like to thank Editage (www.editage.jp) for English language editing.

## DECLARATIONS

### Funding

This study was not funded by any grant.

### Authors’ contributions

Masataka Hirano drafted the manuscript.

Masanori Tsujie contributed to the data analysis and interpretation and assisted in the preparation of the manuscript.

All authors are accountable for all aspects of the work.

### Availability of data and materials

The data that support the findings of this study are not available for public access due to patient privacy concerns but are available on reasonable requests from the corresponding author, Masataka Hirano.

### Ethical approval and consent to participate

The publication of this case report was approved by the Institutional Ethics Committee.

### Consent for publication

The case report and publication process were explained to the patient, who granted permission to publish the report.

### Competing interests

The authors declare that they have no competing interests.
